# Supplementary material for: Protective Efficacy of a Mucosal Influenza Vaccine Formulation Based on the Recombinant Nucleoprotein Co-Administered with a TLR2/6 Agonist BPPcysMPEG
Source: Pharmaceutics. 2023 Mar 10;15(3):912. doi: 10.3390/pharmaceutics15030912 (PMC10057018; doi:10.3390/pharmaceutics15030912)
Supplement: Supplementary file 1 [file pharmaceutics-15-00912-s001.zip › pharmaceutics-2120459-supplementary.pdf]

pharmaceutics

MDPI

Supplementary Materials: Protective Efficacy of a mucosal Influenza Vaccine formulation based on the recombinant nucleoprotein co-administered with a TLR2/6 agonist BPPcysMPEG

Maria Victoria Sanchez, Thomas Ebensen, Kai Schulze, Diego Esteban Cargnelutti, Eduardo A. Scodeller and Carlos A. Guzmán

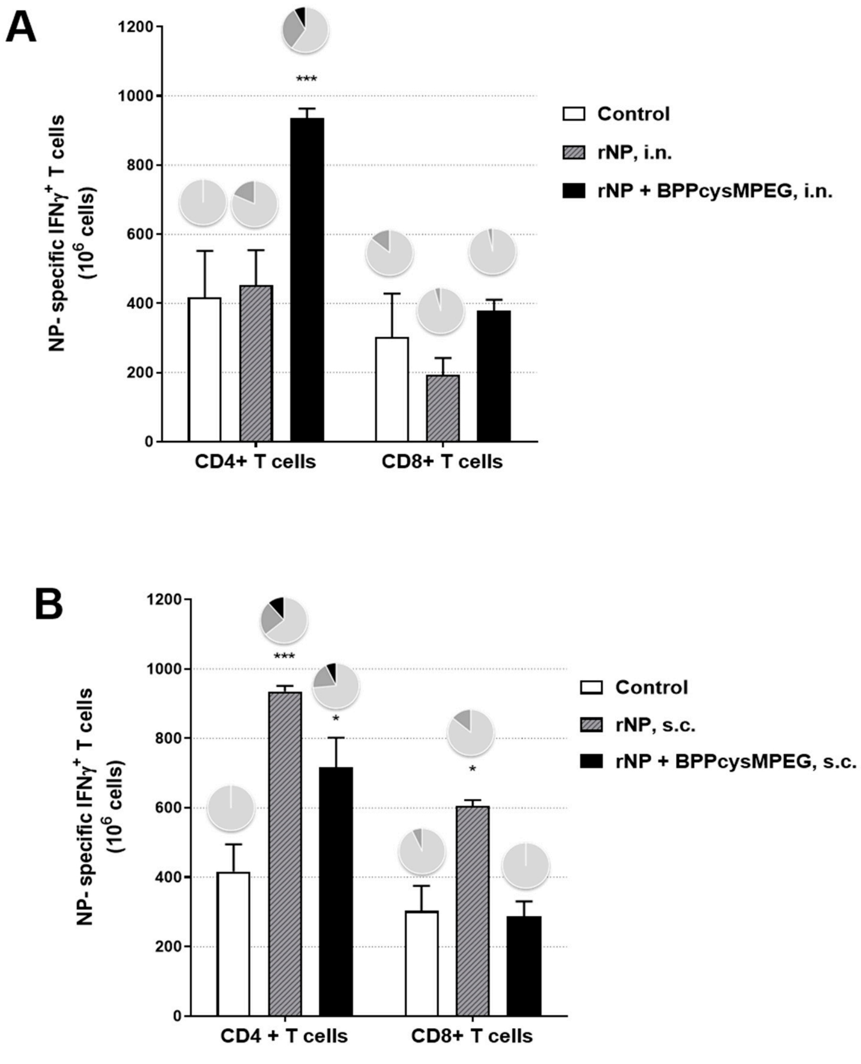

Figure S1. NP-specific multifunctional CD4+ and CD8+ T cell activity. The pie charts show the proportion of single (light grey), double (IFNγ+/TNFα+, IFNγ+/IL-2+; dark grey) and triple (IFNγ+/TNFα+/IL-2+; black) cytokine producers; the bars represent the frequency (mean + SEM) of single IFNγ+ CD4+ and CD8+ T cells derived from mice vaccinated by (**A**) i.n. or (**B**) s.c. route. The number of unstimulated cells was subtracted from the respective number of stimulated cells. Statistical analysis between the adjuvanted and non-adjuvanted groups was performed by two-tailed Student's t-test and two-way ANOVA. Differences were statistically significant (*, $p < 0.1$; *** $p < 0.001$) with respect to values obtained in control mice and/or mice receiving rNP alone.

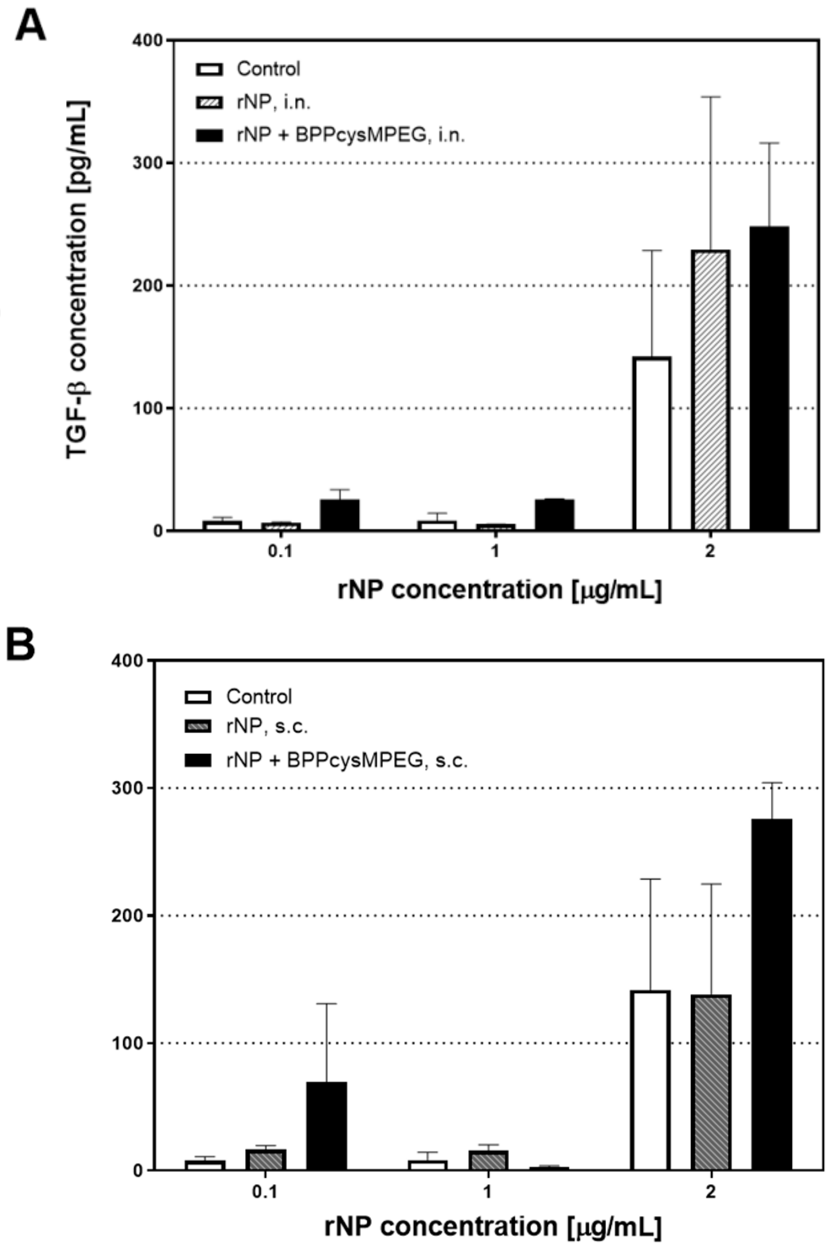

Figure S2. NP-specific TGFß production. The presence of mouse TGF-ß was determined by an ELISA according to the manufacturer's instructions (eBioscience). (**A**) i.n. (**B**) s.c. SEM are indicated by vertical lines.

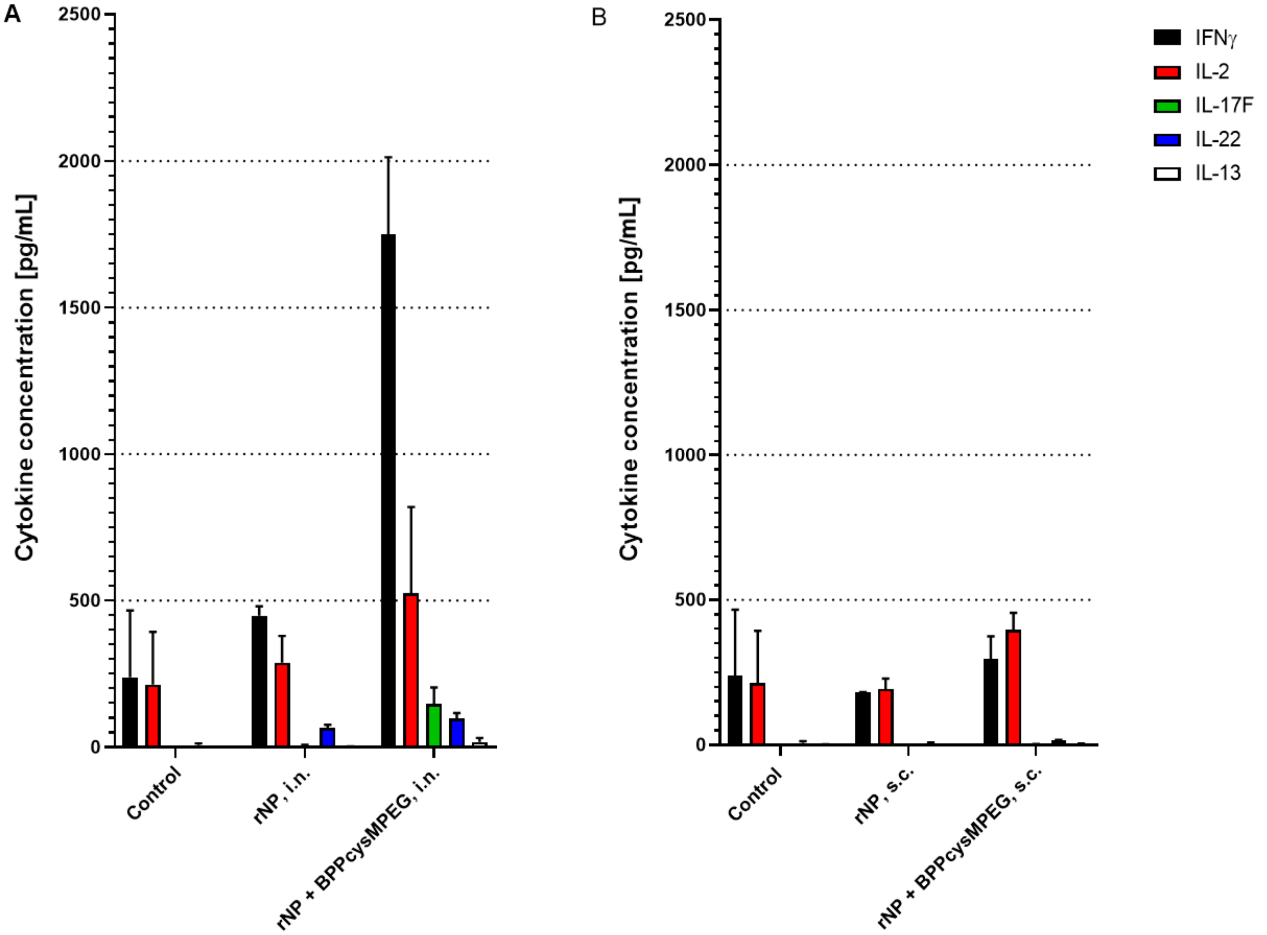

Figure S3. Cytokine profiles antigen-restimulated splenocytes derived from vaccinated mice. The presence of mouse IFN-γ, IL-2, IL-13, IL-17F and IL-22 were determined using a cytometric bead array. Results are presented as cytokine concentration in [pg/mL] of Th1, Th2 and Th17 cytokines secreted by antigen-restimulated splenocytes derived from (**A**) mice vaccinated by i.n. and (**B**) by s.c. route.
